# Procedural volume is linearly associated with mortality, major complications, and readmissions in patients undergoing malignant brain tumor resection

**DOI:** 10.1007/s11060-024-04800-5

**Published:** 2024-09-12

**Authors:** Jane S. Han, Talia Wenger, Alexandra N. Demetriou, Jonathan Dallas, Li Ding, Gabriel Zada, William J. Mack, Frank J. Attenello

**Affiliations:** 1 Department of Neurological Surgery, Keck School of Medicine, University of Southern California, 1200 North State St. Suite 3300, Los Angeles, CA 90033 USA; 2 Department of Population and Public Health Sciences, Keck School of Medicine, University of Southern California, Los Angeles, CA USA

**Keywords:** Malignant brain tumor, Hospital volume, Nationwide readmissions database, Resection

## Abstract

**Purpose:**

Improved outcomes have been noted in patients undergoing malignant brain tumor resection at high-volume centers. Studies have arbitrarily chosen high-volume dichotomous cutoffs and have not evaluated volume-outcome associations at specific institutional procedural volumes. We sought to establish the continuous association of volume with patient outcomes and identify cutoffs significantly associated with mortality, major complications, and readmissions. We hypothesized that a linear volume-outcome relationship can estimate likelihood of adverse outcomes when comparing any two volumes.

**Methods:**

The patient cohort was identified with ICD-10 coding in the Nationwide Readmissions Database(NRD). The association of volume and mortality, major complications, and 30-/90-day readmissions were evaluated in multivariate analyses. Volume was used as a continuous variable with two/three-piece splines, with various knot positions to reflect the best model performance, based on the Quasi Information Criterion(QIC).

**Results:**

From 2016 to 2018, 34,486 patients with malignant brain tumors underwent resection. When volume was analyzed as a continuous variable, mortality risk decreased at a steady rate of OR 0.988 per each additional procedure increase for hospitals with 1–65 cases/year(95% CI 0.982–0.993, p < 0.0001). Risk of major complications decreased from 1 to 41 cases/year(OR 0.983, 95% CI 0.979–0.988, p < 0.0001), 30-day readmissions from 1 to 24 cases/year(OR 0.987, 95% CI 0.979–0.995, p = 0.001) and 90-day readmissions from 1 to 23 cases/year(OR 0.989, 95% CI 0.983–0.995, p = 0.0003) and 24–349 cases/year(OR 0.9994, 95% CI 0.999–1, p = 0.01).

**Conclusion:**

In multivariate analyses, institutional procedural volume remains linearly associated with mortality, major complications, and 30-/90-day readmission up to specific cutoffs. The resulting linear association can be used to calculate relative likelihood of adverse outcomes between any two volumes.

## Introduction

The incidence of malignant brain and central nervous system (CNS) tumors in the United States has been reported as 6.94 per 100,000 individuals, and the average annual mortality associated with these lesions was 4.42 per 100,000 people between 2016 and 2020 [1]. Surgery serves as a mainstay of malignant brain tumor management in many cases, with maximal resection contributing to improved outcomes [2]. It is therefore essential to understand the determinants of post-surgical morbidity and mortality to optimize care delivery for patients with malignant brain tumors.

Numerous patient-specific factors have been previously associated with increased morbidity or mortality following brain tumor resection, including age, race/ethnicity, and medical comorbidities [3, 4]. Similarly, characteristics of the surgeon and treating hospital have also been shown to significantly influence patient outcomes [5]. Specifically, a correlation between higher case volume and improved outcomes has been seen in both primary and metastatic brain tumors and can additionally be observed across various types of procedures in both the CNS and other organ systems [3, 6–8]. That said, existing studies are limited by examining the relationship between case volume and outcomes utilizing groupings such as quartiles or dichotomies determined in a non-systematic fashion [3, 5, 7, 9, 10]. Thus in this present study, we utilize a continuous and additive function to not only better characterize the linear relationship between case volume and patient outcomes, but also systematically identify an inflection point in which this relationship becomes less significant using the Nationwide Readmissions Database (NRD), which to the best of our knowledge has not previously been done in this patient cohort.

## Methods

### Data acquisition

The NRD from the Healthcare Cost and Utilization Project is the largest public national database that provides data on inpatient stays from 28 states regardless of payer option [11]. De-identified patient data can be tracked for one calendar year from the index admission within each state using a linkage number. A retrospective analysis of the NRD from 2016 to 2018 was conducted using International Classification of Diseases, Tenth Edition (ICD-10) codes.

### Study population

Adult patients (≥ 18 years) with primary and/or secondary malignant brain tumor diagnoses that underwent craniotomies were identified using appropriate ICD-10 diagnosis (malignant neoplasm of cerebral meninges, brain, cranial nerve(s), central nervous system: C70.0, C70.9, C71.0–71.9, C72.50, C72.59; secondary malignant neoplasm of brain, cerebral meninges, unspecified/other part of nervous system: C79.31, C79.32, C79.49, C79.40) and procedure codes (brain tumor resection: 00B70ZZ, 00B73ZZ, 00B74ZZ, 00500ZZ, 00503ZZ, 00504ZZ, 00B00ZZ, 00B03ZZ, 00B04ZZ, 00B70ZZ, 00B73ZZ, 00B74ZZ, 00510ZZ, 00513ZZ, 00514ZZ, 00B10ZZ, 00B13ZZ, 00B14ZZ).

### Patient, hospital and clinical characteristics

Patient demographics included: age in quartiles, sex, primary payer, and median household income in quartiles. Hospital characteristics included: ownership, bed size, teaching hospital status, and urban–rural status. Clinical variables included: All patients refined diagnosis related groups (APR-DRG) risk of mortality or disease severity, and Elixhauser comorbidity score. APR-DRG scoring methodology is a standardized scoring system developed by 3 M estimating patient likelihood of mortality or disease severity, classified as minor, moderate, major, or extreme [12]. The system has been utilized by prior studies to adjust for likelihood of mortality or adverse events in multivariate analysis. Agency for Healthcare Research and Quality’s software was used to calculate the Elixhauser comorbidity score. The categorical details and associated descriptive statistics for these variables can be seen in Table 1. Comorbidity diagnoses were queried using ICD-10 diagnosis codes. Outcomes evaluated include in-hospital mortality, major complications, discharge disposition, and readmission at 30-and 90-days. Major complications included pneumonia, pulmonary embolism, renal failure, cerebrovascular accident, myocardial infarction, cardiac arrest, sepsis, and septic shock. This set of major complications was adopted from prior NRD manuscripts evaluating complications associated with surgical and neurosurgical procedures [13, 14]. Hospital procedure volume (as a linear variable) was defined as frequency of tumor resection performed for malignant brain tumor admissions within the calendar year.

**Table 1:** Patient and hospital demographics of patients with malignant brain tumors undergoing resection categorized by hospital procedure volume

	Procedure volume				p-value
Variable	1–2	3–6	7–16	17–349	
Total	632 (1.83%)	2201 (6.38%)	5674 (16.45%)	25,979 (75.33%)	
Age, years					< 0.0001
18–44	49 (7.75%)	228 (10.36%)	583 (10.27%)	4493 (17.29%)	
45–59	190 (30.06%)	632 (28.71%)	1606 (28.3%)	7913 (30.46%)	
60–74	282 (44.62%)	995 (45.21%)	2614 (46.07%)	10,618 (40.87%)	
75 +	111 (17.56%)	346 (15.72%)	871 (15.35%)	2955 (11.37%)	
Sex					0.11
Male	333 (52.69%)	1154 (52.43%)	3001 (52.89%)	14,099 (54.27%)	
Female	299 (47.31%)	1047 (47.57%)	2673 (47.11%)	11,880 (45.73%)	
Primary Payer					< 0.0001
Medicare	287 (45.41%)	1038 (47.16%)	2637 (46.48%)	10,026 (38.59%)	
Medicaid	107 (16.93%)	327 (14.86%)	745 (13.13%)	2661 (10.24%)	
Private insurance	218 (34.49%)	713 (32.39%)	2006 (35.35%)	11,969 (46.07%)	
Other	17 (2.69%)	121 (5.5%)	284 (5.01%)	1277 (4.92%)	
Missing	*DS	*DS	*DS	46 (0.18%)	
APR-DRG: Risk of Mortality Subclass					< 0.0001
Minor	87 (13.77%)	354 (16.08%)	923 (16.27%)	4947 (19.04%)	
Moderate	249 (39.4%)	702 (31.89%)	1880 (33.13%)	8019 (30.87%)	
Major	212 (33.54%)	803 (36.48%)	2051 (36.15%)	8988 (34.6%)	
Extreme	84 (13.29%)	342 (15.54%)	820 (14.45%)	4025 (15.49%)	
Elixhauser Index					< 0.0001
0	23 (3.64%)	119 (5.41%)	340 (5.99%)	2393 (9.21%)	
1	70 (11.08%)	344 (15.63%)	915 (16.13%)	5530 (21.29%)	
2	122 (19.3%)	435 (19.76%)	1187 (20.92%)	5844 (22.5%)	
≥ 3	417 (65.98%)	1303 (59.2%)	3232 (56.96%)	12,212 (47.01%)	
Median Household Income					< 0.0001
0-25th percentile	173 (27.37%)	570 (25.9%)	1303 (22.96%)	4721 (18.17%)	
26th to 50th percentile	172 (27.22%)	626 (28.44%)	1459 (25.71%)	5877 (22.62%)	
51st to 75th percentile	153 (24.21%)	520 (23.63%)	1483 (26.14%)	6805 (26.19%)	
76th to 100th percentile	122 (19.3%)	455 (20.67%)	1339 (23.6%)	8175 (31.47%)	
Missing	12 (1.9%)	30 (1.36%)	90 (1.59%)	401 (1.54%)	
Control/Ownership of Hospital					0.0002
Government, nonfederal	68 (10.76%)	247 (11.22%)	711 (12.53%)	3587 (13.81%)	
Private	564 (89.24%)	1954 (88.78%)	4963 (87.47%)	22,392 (86.19%)	
Bed Size of Hospital					< 0.0001
Small	166 (26.27%)	311 (14.13%)	555 (9.78%)	744 (2.86%)	
Medium	240 (37.97%)	879 (39.94%)	1776 (31.3%)	2975 (11.45%)	
Large	226 (35.76%)	1011 (45.93%)	3343 (58.92%)	22,260 (85.68%)	
Teaching Hospital					< 0.0001
Yes	303 (47.94%)	811 (36.85%)	1375 (24.23%)	1086 (4.18%)	
No	329 (52.06%)	1390 (63.15%)	4299 (75.77%)	24,893 (95.82%)	
Patient Location: NCHS Urban–Rural Code					< 0.0001
Metro	351 (55.54%)	1201 (54.57%)	3008 (53.01%)	19,783 (76.15%)	
Other	281 (44.46%)	1000 (45.43%)	2666 (46.99%)	6196 (23.85%)	
Admission Type					< 0.0001
Missing	*DS	*DS	*DS	25 (0.1%)	
Non-elective	436 (68.99%)	1485 (66.24%)	3669 (64.66%)	13,620 (52.43%)	
Elective	195 (30.85%)	737 (33.48%)	1997 (35.20%)	12,334 (47.48%)	
HCUP Emergency Department Service Indicator					< 0.0001
No	286 (45.25%)	977 (44.39%)	2832 (49.91%)	16,804 (64.68%)	
Yes	346 (54.75%)	1224 (55.61)	2842 (50.09%)	9175 (35.32%)	
Died During Hospitalization					< 0.0001
No	616 (97.47%)	2149 (97.64%)	5559 (97.97%)	25,657 (98.76%)	
Yes	16 (2.53%)	52 (2.36%)	115 (2.03%)	322 (1.24%)	
Major Complications					< 0.0001
No	556 (87.97%)	1940 (88.14%)	5102 (89.92%)	24,085 (92.71%)	
Yes	76 (12.03%)	261 (11.86%)	572 (10.08%)	1894 (7.29%)	
Discharge Disposition					< 0.0001
Home	313 (49.53%)	1056 (47.98%)	2715 (47.85%)	10,894 (41.93%)	
Other	303 (47.94%)	1093 (49.66%)	2843 (50.11%)	14,761 (56.82%)	
Missing	16 (2.53%)	52 (2.36%)	116 (2.04%)	324 (1.25%)	
30-day Readmission					0.008
Yes	96 (16.67%)	324 (16.18%)	769 (14.83%)	3319 (13.97%)	
No	480 (83.33%)	1678 (83.82%)	4416 (85.17%)	20,447 (86.03%)	
90-day Readmission					< 0.0001
Yes	166 (34.02%)	544 (32.95%)	1319 (31.42%)	5321 (27.21%)	
No	322 (65.98%)	1107 (67.05%)	2879 (68.58%)	14,236 (72.79%)	

*APR-DRG* All Patients Refined Diagnosis Related Groups

*NCHS* National Center for Health Statistics

*HCUP* Healthcare Cost and Utilization Project

*DS Data Suppressed

### Statistical analysis

Variables (patient demographic, clinical, hospital) were reported as frequency and percentage with Chi-square test across procedure volume quartiles. Multivariable logistic regression was used to evaluate the relationship between procedure volume and outcomes of in-hospital mortality, major complication, discharge disposition, 30-day, and 90-day readmission. The generalized estimation equation was applied to all models to account for the hospital clustering effect. Variables included in the model are procedure volume, age, gender, primary payer, APR-DRG disease severity, Elixhauser index, income quartiles, hospital ownership, hospital bed size, teaching hospital status, and patient location. Procedure volume was treated as a continuous variable. First, the relationship between procedure volume and outcomes was explored by the Locally estimated scatterplot smoothing (LOESS) with logit transformation of the outcome. Then, we tested procedure volumes with fractional polynomial transformations (degree-1 and degree-2) and splines. The fractional polynomials include powers among − 2, − 1, − 0.5, 0, 0.5, 1, 2, 3. For the splines, the number of pieces and the initial knot location were guided by the LOESS plot. We then tested from − 7 to + 7 of the initial location by an increment of 1. Finally, the final model for each outcome was selected from all fractional polynomial transformations and splines with the lowest Quasi-likelihood under the Independence Model Criterion. Our final models are two-piece splines for in-hospital mortality (knot at 65), major complication (knot at 41), 90-day readmission (knot at 23); three-piece splines for 30-day readmission (knots at 24 and 57); linear procedure volume with discharge disposition. Significance was defined as a 2-sided p value $\le$ 0.05. All analyses were performed using SAS 9.4 (SAS Institute Inc., Cary, NC, USA.).

## Results

### Patient characteristics

A total of 34,486 patients with primary and/or secondary malignant brain tumors underwent resection from 2016 to 2018. The majority of the patients (n = 25,979, 75.3%) underwent resection at hospitals that performed at least 17 cases per year (75th percentile). Irrespective of volume, most patients were at least 60 years of age (1–2: 62.2%; 3–6: 60.9%; 7–16: 61.4; 17–349: 52.2%), male (1–2: 52.7%; 3–6: 52.4%; 7–16: 52.9; 17–349: 54.3%), and had an Elixhauser comorbidity index score of at least 3 (1–2: 65.98%; 3–6: 59.2%; 7–16: 59.96; 17–349: 47.0%). Patients treated at hospitals in the top 75th percentile were more likely to be insured by private insurance (46.1%), while the rest were more likely to be insured by Medicare. Patients were more likely to be admitted non-electively at lower volume (< 17) than higher volume hospitals (65–69% vs 52%). On unadjusted analysis, risk of mortality, major complications, 30-day and 90-day readmission were the lowest in the top 75th percentile. These findings are summarized in Table 1.

### Continuous association of hospital volume and patient outcomes

**Fig. 1:**
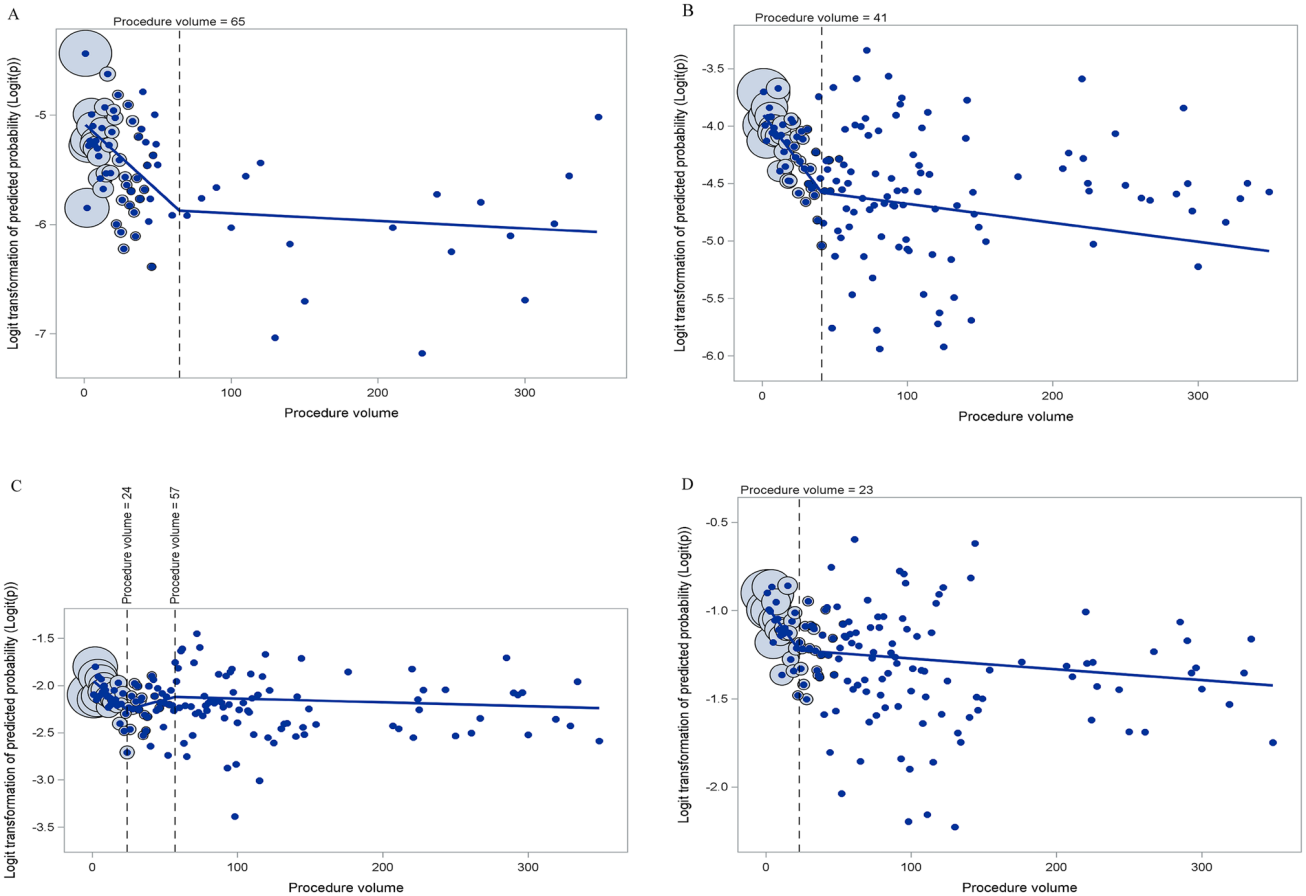
Scatter plot of observed probability versus predicted probability for **a** mortality, **b** major complications, **c** 30-day and **d** 90-day readmissions. **a** For mortality, the predicted probability was calculated from the multivariate regression model, using malignant brain tumor resection volume as two-piece splines connected at 65 cases/year. **b** For major complications, the predicted probability was calculated from the multivariate regression model, using malignant brain tumor resection volume as two-piece splines connected at 41 cases/year. **c** For 30-day readmissions, the predicted probability was calculated from the multivariate regression model, using malignant brain tumor resection volume as three-piece splines connected at 24 cases/year and 57 cases/year. **d** For 90-day readmissions, the predicted probability was calculated from the multivariate regression model, using malignant brain tumor resection volume as two-piece splines connected at 23 cases/year for 90-day readmissions. The area of circles (represented with a black outline) reflects the number of hospitals per procedure volume (represented as circles with a blue outline)

Multivariate analysis of hospital resection volume as a continuous variable with in-hospital mortality exhibited that increase in surgical volume was associated with statistically significant decrease in mortality. Increase in institutional volume from 1 to 65 cases/year was linearly associated (per 1 procedure increase) with decreased mortality (OR 0.988, 95% CI 0.982–0.993, p < 0.0001) (Fig. 1a, Table 2). Linear increase in procedure volume also demonstrated a statistically significant association with decreased major complications. Specifically, for each additional procedure from 1 to 41 cases per year, an association was seen with decreased major complication (OR 0.983, 95% CI 0.979–0.988, p < 0.0001) (Fig. 1b, Table 3). Renal failure and pneumonia were the most common complications in all volume groups, followed by pulmonary embolism and sepsis. Notably, renal failure and pneumonia were significantly more common in the lower volume cohorts (p < 0.0001) (Table 4).

**Table 2:** Factors associated with mortality in patients undergoing surgical resection for malignant brain tumors

Variable	OR (95% CI)	p-value
Procedure Volume		
1–65 (Per 1 increase)	0.988 (0.982–0.993)	** < .0001**
66–349 (Per 1 increase)	0.999 (0.997–1.002)	0.54
Age, years		
18–44	Ref	
45–59	0.74 (0.51–1.07)	0.11
60–74	1.01 (0.72–1.44)	0.94
75 +	1.26 (0.85–1.86)	0.26
Sex		
Male	Ref	
Female	0.98 (0.81–1.18)	0.80
Primary Payer		
Medicare	0.75 (0.53–1.06)	0.10
Medicaid	Ref	
Private insurance	0.81 (0.59–1.11)	0.18
Other	1.42 (0.88–2.28)	0.15
APR-DRG^1^: Risk of Mortality Subclass		
Minor	Ref	
Moderate	1.33 (0.52–3.39)	0.55
Major	3.34 (1.41–7.92)	**0.0063**
Extreme	46 (19.95–106.08)	** < .0001**
Elixhauser Index		
0	Ref	
1	2.4 (0.55–10.54)	0.25
2	4.47 (1.07–18.63)	**0.0396**
≥ 3	8.63 (2.11–35.29)	**0.0027**
Median Household Income		
0–25th percentile	Ref	
26th to 50th percentile	0.9 (0.69–1.16)	0.41
51st to 75th percentile	0.85 (0.65–1.11)	0.23
76th to 100th percentile	0.99 (0.75–1.29)	0.91
Control/Ownership of Hospital		
Government, nonfederal	Ref	
Private	0.87 (0.66–1.15)	0.32
Bed Size of Hospital		
Small	1.17 (0.76–1.8)	0.48
Medium	1.09 (0.85–1.41)	0.5
Large	Ref	
Teaching Hospital		
Yes	Ref	
No	0.96 (0.71–1.3)	0.79
Patient Location: NCHS Urban–Rural Code		
Metro	Ref	
Other	1.25 (1.01–1.55)	**0.041**

*APR-DRG* All Patients Refined Diagnosis Related Groups

*NCHS* National Center for Health Statistics

p < =0.05

**Table 3:** Factors associated with major complications in patients undergoing surgical resection for malignant brain tumors

Variable	OR (95% CI)	p-value
Procedure Volume		
1–41 (Per 1 increase)	0.983 (0.979–0.988)	** < .0001**
42–349 (Per 1 increase)	0.9996 (0.999–1.001)	0.44
Age, years		
18–44	Ref	
45–59	1.2 (0.99–1.46)	0.070
60–74	1.66 (1.37–2.02)	** < .0001**
75 +	2.12 (1.7–2.64)	** < .0001**
Sex		
Male	Ref	
Female	0.63 (0.58–0.69)	** < .0001**
Primary Payer		
Medicare	0.86 (0.73–1.02)	0.078
Medicaid	Ref	
Private insurance	0.9 (0.77–1.06)	0.20
Other	1 (0.79–1.28)	0.98
APR-DRG: Disease Severity		
Minor	Ref	
Moderate	3.36 (1.24–9.09)	**0.017**
Major	19.06 (7.11–51.09)	** < .0001**
Extreme	65.48 (24.46–175.32)	** < .0001**
Elixhauser Index		
0	Ref	
1	2.52 (1.41–4.5)	**0.0018**
2	4.43 (2.5–7.86)	** < .0001**
≥ 3	12.18 (7.02–21.12)	** < .0001**
Median Household Income		
0-25th percentile	Ref	
26th to 50th percentile	0.92 (0.81–1.04)	0.18
51st to 75th percentile	0.87 (0.77–1)	**0.047**
76th to 100th percentile	0.74 (0.65–0.85)	** < .0001**
Control/Ownership of Hospital		
Government, nonfederal	Ref	
Private	1.04 (0.91–1.2)	0.55
Bed Size of Hospital		
Small	0.81 (0.61–1.08)	0.14
Medium	0.92 (0.79–1.07)	0.30
Large	Ref	
Teaching Hospital		
Yes	Ref	
No	1.11 (0.95–1.31)	0.20
Patient Location: NCHS Urban–Rural Code		
Metro	Ref	
Other	0.87 (0.77–0.97)	**0.016**

*APR-DRG* All Patients Refined Diagnosis Related Groups

*NCHS* National Center for Health Statistics

p < =0.05

**Table 4:** Rates of major complications

Major Complications	Procedure Volume				p-value
	**1–2**	**3–6**	**7–16**	**17–349**	
Pneumonia					** < 0.0001**
No	607 (96.04%)	2090 (94.96%)	5456 (96.16%)	25,295 (97.37%)	
Yes	25 (3.96%)	111 (5.04%)	218 (3.84%)	684 (2.63%)	
Pulmonary Embolism					0.54
No	623 (98.58%)	2169 (98.55%)	5609 (98.85%)	25,684 (98.86%)	
Yes	9 (1.42%)	32 (1.45%)	65 (1.15%)	295 (1.14%)	
Renal Failure					** < 0.0001**
No	593 (93.83%)	2074 (94.23%)	5358 (94.43%)	25,030 (96.35%)	
Yes	39 (6.17%)	127 (5.77%)	316 (5.57%)	949 (3.65%)	
Cerebrovascular Accident					0.64
No	632 (100%)	2201 (100%)	5671 (99.95%)	25,970 (99.97%)	
Yes	0 (0%)	0 (0%)	3 (0.05%)	9 (0.03%)	
Myocardial Infarction					**0.04**
No	629 (99.53%)	2186 (99.32%)	5645 (99.49%)	25,890 (99.66%)	
Yes	3 (0.47%)	15 (0.68%)	29 (0.51%)	89 (0.34%)	
Cardiac Arrest					**0.04**
No	630 (99.68%)	2196 (99.77%)	5658 (99.72%)	25,943 (99.86%)	
Yes	2 (0.32%)	5 (0.23%)	16 (0.28%)	36 (0.14%)	
Sepsis					0.38
No	624 (98.73%)	2176 (98.86%)	5614 (98.94%)	25,747 (99.11%)	
Yes	8 (1.27%)	25 (1.14%)	60 (1.06%)	232 (0.89%)	
Septic Shock					0.55
No	629 (99.53%)	2192 (99.59%)	5658 (99.72%)	25,908 (99.73%)	
Yes	3 (0.47%)	9 (0.41%)	16 (0.28%)	71 (0.27%)	

p < =0.05

Additive increase in procedural volume was also significantly associated with decrease in unplanned 30-day and 90-day readmission in multivariate analysis. Specifically, likelihood of 30-day readmission decreased in an additive fashion from 1–24 cases per year (OR 0.987, 95% CI 0.979–0.995, p = 0.001) (Fig. 1c, Table 5) and likelihood of 90-day readmission decreased from 1 to 23 cases (OR 0.989, 95% CI 0.983–0.995, p = 0.0003) and 24–349 cases (OR 0.9994, 95% CI 0.999–1, p = 0.01) (Fig. 1d, Table 6). The downward trend association between increasing volume and decreasing adverse outcomes continues at higher volumes for all outcomes after 349 cases, but levels off and becomes less pronounced.

**Table 5:** Factors associated with 30-day readmissions in patients undergoing surgical resection for malignant brain tumors

Variable	OR (95% CI)	p-value
Procedure Volume		
1–24 (Per 1 increase)	0.987 (0.979–0.995)	**0.001**
25–57 (Per 1 increase)	1.004 (1–1.008)	**0.03**
58–349 (Per 1 increase)	0.9996 (0.999–1.0003)	0.25
Age, years		
18–44	Ref	
45–59	1.06 (0.95–1.18)	0.29
60–74	1.05 (0.93–1.18)	0.44
75 +	1.02 (0.88–1.2)	0.77
Sex		
Male	Ref	
Female	0.83 (0.78–0.89)	** < .0001**
Primary Payer		
Medicare	0.94 (0.84–1.06)	0.29
Medicaid	Ref	
Private insurance	0.8 (0.72–0.89)	** < .0001**
Other	0.76 (0.64–0.9)	**0.0016**
APR-DRG: Disease Severity		
Minor	Ref	
Moderate	1.16 (1.04–1.29)	**0.0082**
Major	1.5 (1.34–1.67)	** < .0001**
Extreme	1.53 (1.35–1.74)	** < .0001**
Elixhauser Index		
0	Ref	
1	1.06 (0.9–1.24)	0.48
2	1.29 (1.1–1.5)	**0.0015**
≥ 3	1.71 (1.48–1.98)	** < .0001**
Median Household Income		
0-25th percentile	Ref	
26th to 50th percentile	0.88 (0.8–0.97)	**0.0072**
51st to 75th percentile	0.92 (0.83–1.01)	0.086
76th to 100th percentile	0.91 (0.82–1)	0.057
Control/Ownership of Hospital		
Government, nonfederal	Ref	
Private	1.06 (0.95–1.18)	0.31
Bed Size of Hospital		
Small	0.78 (0.64–0.96)	**0.022**
Medium	0.97 (0.87–1.08)	0.61
Large	Ref	
Teaching Hospital		
Yes	Ref	
No	0.88 (0.77–1.01)	0.0595
Patient Location: NCHS Urban–Rural Code		
Metro	Ref	
Other	0.89 (0.82–0.97)	**0.0055**

*APR-DRG* All Patients Refined Diagnosis Related Groups

*NCHS* National Center for Health Statistics

p < =0.05

**Table 6:** Factors associated with 90-day readmissions in patients undergoing surgical resection for malignant brain tumors

Variable	OR (95% CI)	p-value
Procedure Volume		
1–23 (Per 1 increase)	0.989 (0.983–0.995)	**0.0003**
24–349 (Per 1 increase)	0.9994 (0.999–1)	**0.01**
Age, years		
18–44	Ref	
45–59	1.22 (1.11–1.33)	** < .0001**
60–74	1.26 (1.14–1.4)	** < .0001**
75 +	1.22 (1.06–1.41)	**0.0055**
Sex		
Male	Ref	
Female	0.88 (0.84–0.93)	** < .0001**
Primary Payer		
Medicare	0.9 (0.8–1)	**0.047**
Medicaid	Ref	
Private insurance	0.74 (0.68–0.81)	** < .0001**
Other	0.73 (0.63–0.85)	** < .0001**
APR-DRG: Disease Severity		
Minor	Ref	
Moderate	1.28 (1.16–1.4)	** < .0001**
Major	1.52 (1.39–1.67)	** < .0001**
Extreme	1.53 (1.37–1.71)	** < .0001**
Elixhauser Index		
0	Ref	
1	1.21 (1.07–1.37)	**0.0025**
2	1.39 (1.22–1.58)	** < .0001**
≥ 3	1.81 (1.59–2.06)	** < .0001**
Median Household Income		
0-25th percentile	Ref	
26th to 50th percentile	0.93 (0.85–1.01)	0.074
51st to 75th percentile	0.92 (0.85–1.01)	0.075
76th to 100th percentile	0.91 (0.83–1)	**0.045**
Control/Ownership of Hospital		
Government, nonfederal	Ref	
Private	1.04 (0.95–1.15)	0.37
Bed Size of Hospital		
Small	0.91 (0.74–1.11)	0.34
Medium	0.95 (0.86–1.05)	0.32
Large	Ref	
Teaching Hospital		
Yes	Ref	
No	0.93 (0.83–1.04)	0.20
Patient Location: NCHS Urban–Rural Code		
Metro	Ref	
Other	0.9 (0.83–0.97)	**0.0036**

*APR-DRG* All Patients Refined Diagnosis Related Groups

*NCHS* National Center for Health Statistics

p < =0.05

To provide readers with estimates of the additive effect of improvement in patient outcomes between differing fixed institutional volumes, we generated tables comparing relative risk at various procedure volumes of from 1 to 349 cases (Tables 7, 8, 9, 10). The association of increased institutional volume with decreased adverse outcomes was still noted when we estimated mortality, major complications, 30-and 90-day readmission risks at different volume thresholds (Fig. 1a–d). As seen in our figures, the amplitude of change does decrease at higher volume thresholds, but the benefit does continue to increase significantly with volume in a non-linear fashion throughout the entire range. Using these tables, the likelihood of adverse outcomes between differing institutional volumes may be estimated.

**Table 7:** Estimated risk of mortality at different volume thresholds

Procedure Volume	OR	95% CI	p-value
1	Ref		
10	0.896	0.852—0.942	< 0.0001
30	0.701	0.596—0.823	< 0.0001
50	0.548	0.418—0.720	< 0.0001
65	0.456	0.320—0.651	< 0.0001
70	0.450	0.314—0.643	< 0.0001
100	0.440	0.316—0.614	< 0.0001
200	0.411	0.293—0.578	< 0.0001
300	0.384	0.241—0.611	< 0.0001
349	0.371	0.214—0.642	0.0004

**Table 8:** Estimated risk of major complications at different volume thresholds

Procedure Volume	OR	95% CI	p-value
1	Ref		
10	0.859	0.823—0.897	< 0.0001
30	0.613	0.533—0.705	< 0.0001
41	0.509	0.420—0.617	< 0.0001
50	0.499	0.411—0.606	< 0.0001
100	0.490	0.408—0.589	< 0.0001
200	0.473	0.389—0.575	< 0.0001
300	0.456	0.357—0.582	< 0.0001
349	0.448	0.339—0.591	< 0.0001

**Table 9:** Estimated risk of 30-day readmissions at different volume thresholds

Procedure Volume	OR	95% CI	p-value
1	Ref		
10	0.888	0.827—0.953	0.001
24	0.738	0.615—0.884	0.001
40	0.775	0.658—0.913	0.0022
57	0.832	0.712—0.972	0.0205
100	0.821	0.705—0.957	0.0115
200	0.788	0.667—0.932	0.0053
300	0.756	0.615—0.930	0.0079
349	0.741	0.589—0.934	0.011

**Table 10:** Estimated risk of 90-day readmissions at different volume thresholds

Procedure Volume	OR	95% CI	p-value
1	Ref		
10	0.905	0.857—0.955	0.0003
23	0.783	0.685—0.894	0.0003
50	0.762	0.664—0.873	< 0.0001
100	0.739	0.645—0.846	< 0.0001
200	0.695	0.601—0.804	< 0.0001
300	0.654	0.552—0.774	< 0.0001
349	0.635	0.528—0.762	< 0.0001

Other factors, such as higher APR-DRG risk of mortality score and higher Elixhauser comorbidity score were also independently associated with increased likelihood of mortality, major complications, 30- and 90-day readmissions. Age ≥ 60 was associated with a higher likelihood of major complications and ≥ 45 was associated with a higher likelihood of 30-day readmissions. Further, female sex was associated with decreased likelihood of major complications, 30-day, and 90-day readmissions. These findings are summarized in Tables 2, 3, 5, 6.

## Discussion

Using the Nationwide Readmissions Database, we quantitatively and systematically examined the relationship between hospital procedure volume as a linear variable and outcomes in patients undergoing craniotomies for malignant primary or secondary brain tumors. We conducted a continuous volume-outcome multivariate analysis and demonstrated that a significant linear association between increasing institutional volume and patient mortality, major complications, 30-day and 90-day readmissions. After the very high institutional volumes, the effect size of the volume-outcome relationship becomes less pronounced but remains significant. Further, we estimated the risk of mortality, major complications, 30-day and 90-day readmission after resection at respective volume thresholds throughout the reported range of volume. This confirmed that the risk of each outcome improved with volume increase in an additive fashion and can be utilized to compare the risk of each outcome of different institutions at any comparative volume. To our knowledge, prior studies have only confirmed the volume-outcome relationship in this patient cohort by arbitrarily dichotomizing hospital volume into high and low cohorts and our group is the first to use a national database to conduct a multivariate linear analysis to investigate the impact of procedural volume on patient outcomes in a continuous and additive function in this patient cohort [3, 6, 9, 10, 15].

In our multivariate analysis of institutional procedure volume as a continuous variable, we showed that volume was significantly associated with increased likelihood of mortality, major complications, 30-day and 90-day readmissions in an additive fashion. Though prior studies do not evaluate the association between volume and adverse outcome using procedural volume as a linear variable, these studies similarly demonstrated a significant association between high institutional procedural volume and decreased risk of poor outcomes across multiple surgical subspecialties [10, 15–21]. Among analyses of craniotomies for brain tumors, high-volume hospitals have repeatedly been found to be associated with a decreased likelihood of mortality, major complications, and/or adverse discharge disposition [3, 6, 7, 9, 10, 16]. While the relationship between hospital volume and patient outcome clearly exists, the reason for this association is debated [22]. Prior studies have attributed several reasons for this effect, with one explanation being the “practice-makes-perfect” theory, which presumes that surgeons at high-volume institutions have more opportunity to practice their surgical technique for particular procedures, and are thus more experienced in handling complications and dealing with anomalous anatomy [10, 16, 23, 24]. In the United Kingdom, the National Institute for Health and Care Excellence recommends that neurosurgeons practicing surgical neuro-oncology commit at least half of their practice to treating neuro-oncology patients [7]. On a hospital/systems level, increased accessibility of resources, such as specialized multidisciplinary teams (e.g. neuroradiologists, neuro-oncologists), neurosurgical intensive care units staffed with dedicated neurologically-trained personnel, and advanced diagnostic or therapeutic technologies may also contribute to improved outcomes at higher volume hospitals [7, 10, 25–27]. Additionally, lower volume hospitals may be at a disadvantage of having a higher proportion of non-elective cases which can limit preoperative risk optimization and surgical planning, thus contributing to worsened outcomes. Although lower volume hospitals were more likely to have non-elective admission than higher volume hospitals (top 75th percentile) in the present study, the linear association of volume with outcomes persists even into the highest volume hospitals (with low non-elective surgery rate), suggesting that both volume and the non-elective/emergent nature of surgeries may each play a critical role.

Our results add to the prior understanding of the volume-outcome effect. Prior studies in this patient cohort have only shown the relationship between hospital volume and patient outcomes by distinguishing low- and high-volume centers without an evidence-based approach to the specific cutoff [3, 6, 9, 10, 15]. Most of these studies only compared outcomes between the lowest and highest quartile or quintile hospitals [3, 6, 9, 10, 15]. A resulting challenge lies in resulting hypotheses where improved outcomes are significantly associated with institutions performing procedures only over specific volume cutoffs. The present study systematically defined the volume-outcome curve by conducting a multivariate linear analysis and delineated the graded association between patient outcomes and hospital volume. For all outcomes, the continuous volume-outcome curve demonstrated more significant association between linear increase in volume and decreased adverse outcomes during volume increases at lower volumes. Studies using systematic approaches similar to the present study showing that the association between volume and patient outcomes (e.g. mortality, patient safety indicator events) is not dichotomous have been primarily conducted in patients undergoing treatment for neurovascular pathologies (e.g. subarachnoid hemorrhage [SAH], unruptured intracranial aneurysms) [28–31, 33]. These national database studies observed continuous volume-outcome relationships using adjusted linear models [29–33]. Notably, two of these studies show that the effect size for mortality risk is most prominent at lower volumes and becomes attenuated as volume increases, like the findings of the present study [30, 31]. The limiting volume effect at institutions with higher volumes could potentially be explained by the fact that after a certain threshold, expertise and experience do not significantly differ between these institutions and outcomes are more variable on a case-by-case basis. Higher patient complexity and disease severity at higher volume hospitals could be a contributing factor to limiting improving outcomes at higher volumes as well [6]. Moreover, high-volume hospitals may become overburdened if the caseload exceeds the hospitals’ capabilities and thus attenuate the advantages of high-volume hospitals [34]. Taking together the findings of these studies and the present study, the linear graded relationship may be perhaps a more effective way of understanding the volume-outcome relationship rather than establishing volume cutoffs to dichotomously compare high- and low-volume hospitals.

While increasing volume is associated with a more significant decrease in mortality, major complications, readmission rates at lower volumes, we demonstrate that the additive effect for all outcomes remains significant throughout the entire range of volume. This is notable as prior volume-outcome studies in this patient cohort primarily demonstrated that hospitals with procedure volumes in the highest quartile have better outcomes than those in the lowest quartile [3, 6, 9, 10]. Our results suggest that the likelihood of adverse outcomes examined in this study can be estimated and compared between institutions of different volume thresholds throughout the reported range of volume (as demonstrated in Tables 3, 5, 8, 10), rather than only comparing the highest to the lowest volume cohorts. Successful attempts have been made in other neurosurgical pathologies to find an evidence-based approach to compare institutions of different volumes [28–32]. For instance, a national database study on transsphenoidal pituitary tumor resection outcomes estimated that for every ten additional cases, patients were 11% less likely to have iatrogenic panhypopituitarism and 6% less likely to have diabetes insipidus, suggesting an additive effect [32]. A British study on SAH patients demonstrated that mortality risk decreased in an additive fashion for every additional 100 cases [28]. A national database study on patients undergoing treatment for SAH identified a decrease in inpatient mortality and adverse discharge disposition risk from 20 to 100 cases in an additive fashion as well [30]. This study reported the amplitude of change in risk to be higher at lower volumes similar to the present study [30]. Further, our findings highlight the potential benefits of centralizing care for this patient cohort, which has been shown to positively impact outcomes in this complex patient cohort and more extensively in stroke care [9, 35–37]. Finally, our findings suggest continued study of whether patients initially admitted or establishing care at low-volume centers may benefit from referral and transfer to high-volume, comprehensive centers after any necessary acute symptomatic treatment [38, 39].

## Limitations

Utilizing a national database like the NRD for retrospective studies comes with many inherent limitations, including but not limited to selection bias and coding errors. Although ICD-10 codes were utilized to query clinical variables, data entry may be inconsistent, subjective, and inaccurate due to variability in administrative staff as well as transcription and documentation errors. Additionally, we were unable to query critical clinical variables such as individual provider volume. This is a significant limitation given that multiple providers, likely with different expertise levels, are accounted for in determining institutional procedure volume. Other common variables often commonly evaluated but were not available include patient characteristics (e.g. race/ethnicity, preoperative performance score), tumor-related characteristics (e.g. size, location, histology), extent of resection, operating room team characteristics and floor-specific factors, which could have also contributed to the volume-outcome effect. Further, we could not obtain data such as mortality causes which could provide further information on possible disparities amongst the different volume cohorts. There may also be regional bias given that the NRD captures data from only 28 states and patients cannot be tracked across state lines. Further, we are unable to conduct longitudinal analysis such as evaluating 30-day readmission after 11 months and 90-day readmissions after 9 months, as the NRD resets annually and patients cannot be tracked across different calendar years.

## Conclusion

Here, we assessed the linear relationship between hospital procedural volume and patient outcomes in patients with malignant brain tumors undergoing resection. Among patients with primary and secondary malignant brain tumors, there exists a continuous and additive relationship between decreased mortality, major complications, and 30-day and 90-day readmission and increase in hospital volume that is particularly pronounced at lower volumes. Our methods may be broadly applicable to other neurosurgical approaches and beyond to further delineate the volume-outcome relationship. More research is needed to fully delineate the specific factors that contribute to this volume-outcome relationship.

## Data Availability

No datasets were generated or analysed during the current study.
